# The Advantages of Structural Equation Modeling to Address the Complexity of Spatial Reference Learning

**DOI:** 10.3389/fnbeh.2016.00018

**Published:** 2016-02-26

**Authors:** Pedro S. Moreira, Ioannis Sotiropoulos, Joana Silva, Akihiko Takashima, Nuno Sousa, Hugo Leite-Almeida, Patrício S. Costa

**Affiliations:** ^1^Life and Health Sciences Research Institute (ICVS), School of Health Sciences, University of MinhoBraga, Portugal; ^2^ICVS/3B's - PT Government Associate LaboratoryBraga/Guimarães, Portugal; ^3^RIKEN Brain Science Institute, Laboratory for Alzheimers DiseaseSaitama, Japan; ^4^Department of Aging Neurobiology, National Center for Geriatrics and GerontologyOhbu, Japan

**Keywords:** auto-regressive latent trajectories, reference learning, longitudinal assessments

## Abstract

**Background:** Cognitive performance is a complex process influenced by multiple factors. Cognitive assessment in experimental animals is often based on longitudinal datasets analyzed using uni- and multi-variate analyses, that do not account for the temporal dimension of cognitive performance and also do not adequately quantify the relative contribution of individual factors onto the overall behavioral outcome. To circumvent these limitations, we applied an Autoregressive Latent Trajectory (ALT) to analyze the Morris water maze (MWM) test in a complex experimental design involving four factors: stress, age, sex, and genotype. Outcomes were compared with a traditional Mixed-Design Factorial ANOVA (MDF ANOVA).

**Results:** In both the MDF ANOVA and ALT models, sex, and stress had a significant effect on learning throughout the 9 days. However, on the ALT approach, the effects of sex were restricted to the learning growth. Unlike the MDF ANOVA, the ALT model revealed the influence of single factors at each specific learning stage and quantified the cross interactions among them. In addition, ALT allows us to consider the influence of baseline performance, a critical and unsolved problem that frequently yields inaccurate interpretations in the classical ANOVA model.

**Discussion:** Our findings suggest the beneficial use of ALT models in the analysis of complex longitudinal datasets offering a better biological interpretation of the interrelationship of the factors that may influence cognitive performance.

## Introduction

Water mazes have been proven reliable tools to assess different dimensions of learning and memory in rodents (Morris, 1981; D'Hooge and De Deyn, 2001; Sousa et al., 2006; Vorhees and Williams, 2014) as they exhibit high sensitivity to monitor cognitive performance changes due to different manipulations/treatments (Cerqueira et al., 2007; Leite-Almeida et al., 2009, 2012; Sotiropoulos et al., 2015).

The amount of time, or distance, animals need to reach the platform is used as a behavioral readout, while integrated distances can also be used (e.g., error score Sotiropoulos et al., 2015). These readouts aim to assess the temporal dynamics of learning during the sequential experimental days highlighting the importance of learning/memory evolution and growth in water mazes. The aforementioned parameters are analyzed in a sequential and/or temporal fashion, with stepper (negative) slopes being associated with better performances. Traditionally, authors have either used *t*-tests or One- or Two- Way Analyses of Variance (ANOVA) to compare group differences at each time point. Repeated Measures ANOVA and Mixed-Design Factorial ANOVA (between- and within- subjects factors) have also been employed in this context considering only the factor means (Meredith and Tisak, 1990), though the use of these procedures is still limited in the field (Kilkenny et al., 2009). However, the use of these procedures is often misinterpreted as they have associated strict assumptions that are often not met. Particularly, sphericity violations are associated with an increased false-positives' rate. Also, none of the abovementioned statistical analyses properly assess the temporal dimension (growth) of learning/memory performance, which is a core behavioral element of water mazes assessment. These traditional procedures focus on the interpretation of the means, considering differences among individual animals as error variance. Nevertheless, this variance contains information of upmost relevance for the study of change, providing knowledge about individual trajectories. With this information, it is possible to assess whether the baseline influences the evolution throughout time (e.g., does the animals' learning performance in the first session of an experiment influences the learning during the remaining sessions?). In addition, the influence of several factors, known to affect learning and memory, such as aging, sex, anxiety, and environmental stress (Cerqueira et al., 2007; Leite-Almeida et al., 2009, 2012; Sotiropoulos et al., 2015) may not be properly captured using the above mentioned analyses. In fact, these factors may differentially affect particular characteristics of the learning curve, including starting learning performance, acquisition phase and/or learning growth. Lastly, when the baseline performance differs between groups, comparisons based on mean performance values may be misinterpreted.

To overcome the above drawbacks, we have applied an Autoregressive Latent Trajectory (ALT) approach to study spatial reference learning in the Morris water maze (MWM), using a complex set of data obtained from experimental animals of different ages (middle-aged and old), sexes (male and female), environmental conditions (undisturbed and stressed), and genotypes (wild-type vs. P301L-Tau; Sotiropoulos et al., 2011, 2015). ALT combines two distinct structural equation modeling (SEM) procedures: auto-regressive (AR) and latent growth (LGM). On one hand, this approach allows to study how the scores in one measure influences the scores of the one, that follows (e.g., the influence of day 2 on day 3)—the AR model. Simultaneously, the ALT approach enables the study of underlying patterns of trajectory, i.e., by accounting for factor means, variances and measurement error terms, both inter and intra-individual variability are captured—the LGM model.

## Materials and methods

### Experimental subjects and data

**Figure 1:**
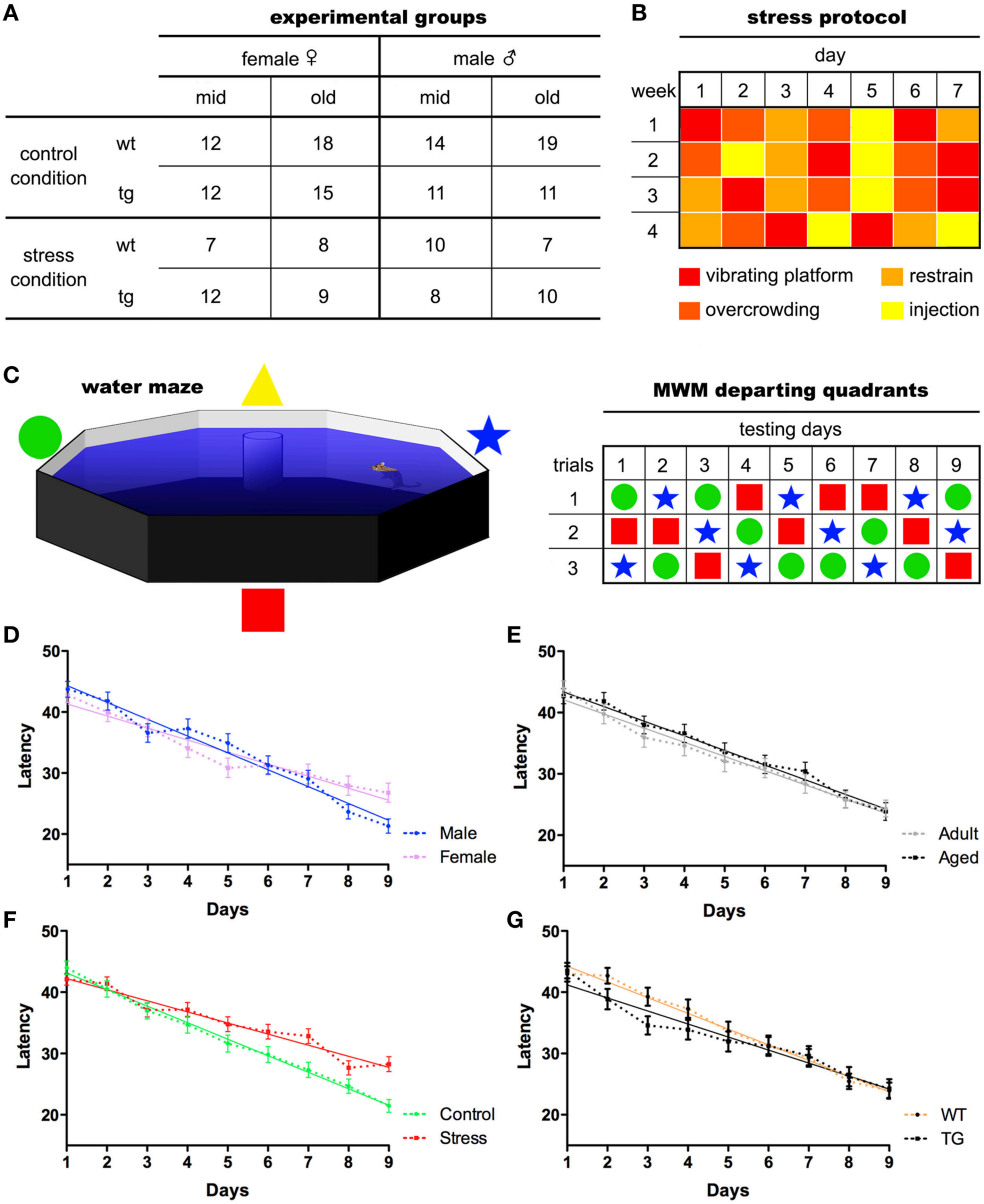
**Experimental organization and main behavioral readouts**. Middle-aged and old mice of both sexes were used in the experiments. The number of experimental subjects ascribed to each group **(A)** is given. Following 28 days of chronic stress paradigm **(B)**, animals performed the Morris water maze test for nine consecutive days, three trials/day, departing from pseudo-randomly assigned pool quadrants **(C)**. Mean latencies were used to assess animals' ability to find the maze platform. All animals learning curves split by sex **(D)**, age **(E)** stress **(F)**, and genotype **(G)** are presented. Mean ± S.E.M.

One-hundred and eighty-three mice of both sexes with different ages, [middle-aged (12–14 months old) and old (22–24 months old)] and genotypes [wild-type (WT) and expressing mutated P301L-Tau [24]] were used (see Figure 1 for details). Mice were housed in groups of four to five animals per cage under standard environmental conditions (ambient temperature 21 ± 1°C; relative humidity of 50–60%; 12 h light/dark cycle, lights on at 8:00 A.M.) with *ad libitum* access to food and water. P301L-Tau and WT animals were randomly assigned to one of two groups: stress and control. Stressed animals were subjected to 28 days of prolonged stress (see protocol below). The behavioral experiments were conducted at the National Center for Geriatrics and Gerontology in Japan, according to Japanese Law. All procedures were approved by the Animal Care and Use Committee of RIKEN institute (Saitama, Japan), and conformed to the US National Institutes of Health Guidelines on animal welfare and experimentation. More information can be found at Sotiropoulos et al. (2015).

### Behavioral procedures

#### Stress protocol

Over a period of 28 days, animals were subjected to four different stressors (one stressor per day) in random order to prevent habituation (Sotiropoulos et al., 2015). These stressors included overcrowding, restraint, placement on a rocking platform and intraperitoneal (i.p.). injection of 0.9% saline 1 ml/100 g (Sotiropoulos et al., 2011; see Figure 1 for details). To analyze the efficiency of the stress protocol, measures of body weight and serum corticosterone levels were obtained (Sotiropoulos et al., 2015).

#### Morris water maze

Animals were tested in a MWM protocol for nine consecutive days as described by Sotiropoulos et al. (Sotiropoulos et al., 2015). The water maze consisted of an opaque cylinder (1 m diameter) filled with water (24°C) placed in a room with reference cues. A transparent escape platform was placed slightly submerged. Learning trials started by gently placing mice on the water surface of the maze. Mice were tested over nine consecutive days (three trials/day—60 s/trial). Swim paths were monitored and recorded by a CCD camera, using Image J software (http://rsb.info.nih.gov/nih-image/). Data were subsequently analyzed using customized software based on Matlab (version 7.2, Mathworks Co Ltd, CA), with an image analysis tool box (Mathworks). The mean latency value of each animal based on the three daily trials performed was used to assess the learning curve.

### Statistical analyses

#### Comparison of analytical procedures

To compare results from longitudinal data using classical and Structural Equation Modeling (SEM) based approaches, a Mixed-Design Factorial (MDF) ANOVA and a hybrid ALT method were employed. The influence of main factors, sex, age, stress, and genotype, and their interacting effects in animals' learning were tested. Although the common practice with ALT method is to integrate all main factors within the same model, we have analyzed each of the main factors and interaction effects in separate models, in order to conduct a direct comparison between procedures. Both AR and LGM are special forms of SEM, employed in a combined manner aiming to explain changes across time as an underlying latent process and with each moment of assessment regressing the following (Duncan and Duncan, 2004).

**Figure 2:**
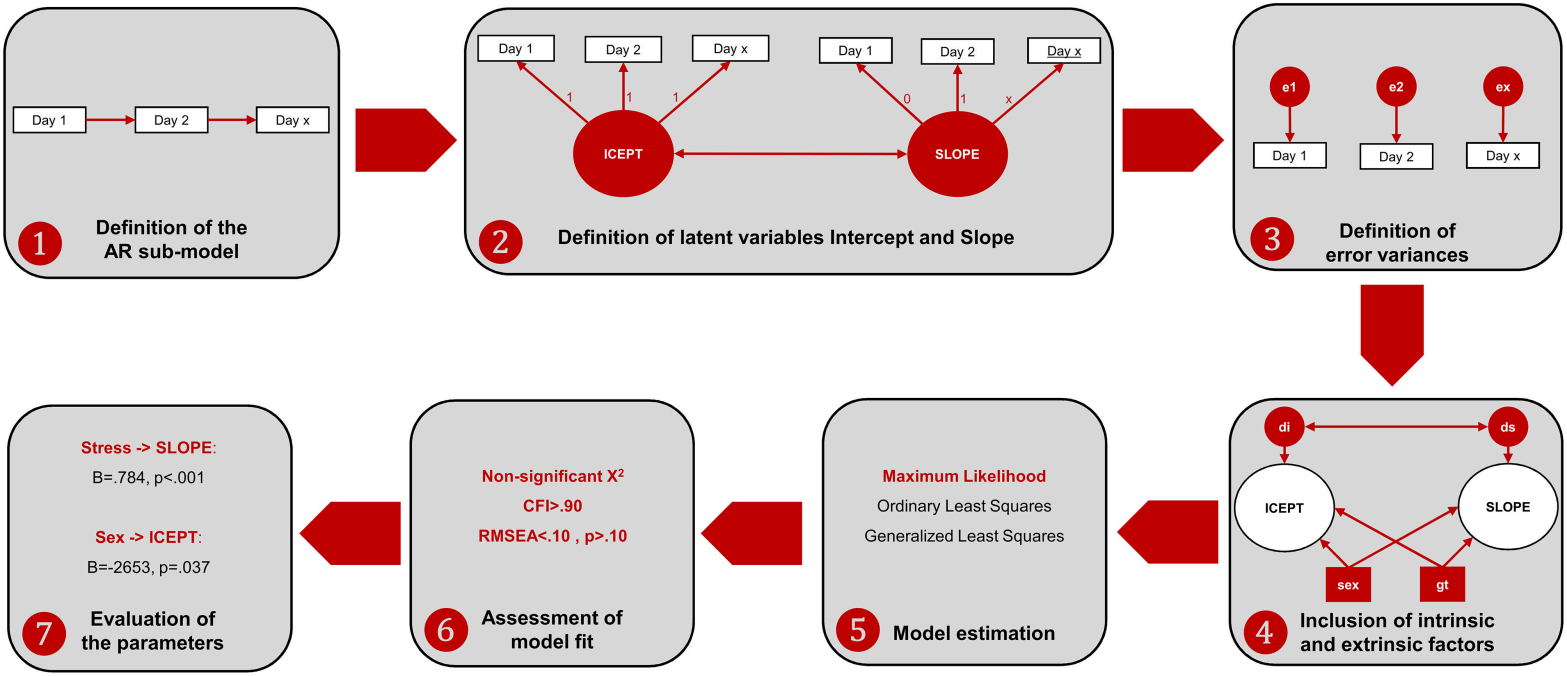
**Workflow of the ALT approach specification and interpretation**. (1) The AR sub-model is established by specifying relationships between consecutive time-points; (2) the variables Intercept and Slope are defined to represent baseline levels (each time-point has the same weight) and linear growth (each time-point has an increase of one-unit comparing to the previous time-point). Additionally, a correlation between Intercept and Slope is established to test whether animals that are better performers at the baseline are those with higher growth learning curves; (3) the variance not explained by neither the intercept nor the slope is specified as error variance (which is also a latent measure); (4) internal and external individual characteristics are included to assess their influence in the intercept and in the slope. Two latent variables are defined to account for the error variance of the intercept (disturbance of the intercept, di) and the slope (disturbance of the slope, ds); (5) the model is estimated with one estimation method (the maximum likelihood is the most often used in the ALT approach); (6) the fit of the model is assessed through the analysis of different indices [as previously mentioned, the chi-square together with incremental (such as the CFI) and another absolute fit indices (such as the RMSEA) should be evaluated]. In the case of poor model fit, the model should be re-specified, making some adjustments (e.g., including non-linear growth variables, such as quadratic or logarithmic growth functions); (7) the parameters are evaluated to assess the relevant associations between variables.

Regarding the MDF ANOVA, it was observed that the assumption of sphericity was violated [$\chi_{\left(35\right)}^{2}=117.62$, *p* < 0.001] and therefore a Hyundt-Felt correction was applied (ε = 0.983). For the ALT approach, the main effect of each factor and all the possible interaction effects on the animals' learning curve were analyzed in individual models. Prior to model specification, the assumption of normality was tested for all the variables, using the following rules-of-thumb: Skewness (*Sk* < 3.0) and Kurtosis (*K* < 8.0). All the variables presented *Sk* and *K* under these reference scores (Kline, 2005). The ALT approach was defined through the specification of AR and LGM sub-models. The AR model was defined by specifying that each time-point is linearly dependent on the previous one (i.e., the performance on 1 day predicts the performance of the next day). The LGM assessed the mean-changes across the different time units (intercept) and the individual variation (slope) in the first time unit. For this purpose, two latent variables^1^ were defined, representing (1) the baseline level (the factor loadings were fixed at 1 for each day of acquisition) and (2) the linear change across time [the loadings were defined in an ascending order (from 0 to 8), representing the different days]. The last step in the model definition was to include intrinsic and extrinsic characteristics (sex, age, stress, and genotype) to test their influence in both the Intercept and the Slope. Afterwards, the parameters of the ALT models (i.e., the pre-defined relationships between variables) were estimated. Goodness-of-fit of the models was evaluated with the χ^2^ statistic and with the following descriptive indices: root mean square error of approximation (RMSEA) and the comparative fit index (CFI; Hu and Bentler, 1999; Schermelleh-Engel et al., 2003)^2^. Figure 2 represents the steps for the specification of ALT models and its interpretation.

#### Integrated ALT approach

An integrated ALT model in which all the factors were entered simultaneously was conducted to assess the combined influence of all factors in the learning curve. Hence, the model accounted for the shared variance between factors. This strategy extends the direct comparison between procedures; it allows to assess which factors affect the learning curve and to calculate the total explained variance for both the baseline (intercept) and the growth (slope) during time. This strategy was not previously applied to animal research experiments.

Descriptive statistics and Mixed-Design Factorial ANOVA were performed with IBM SPSS Statistics v22. The ALT was performed using IBM SPSS AMOS v22.

## Results

### Sample characteristics effects on learning curve

Descriptive statistics of the study variables are presented on Table 1. We found that latencies decreased in linear trend during the nine MWM acquisition days (Figure 1) indicating increased task-solving efficiency across sessions. Even though female and stressed groups started with similar performance to males and non-stressed, respectively (Figure 1), their gain was progressively diminished throughout sessions. In addition, the genotype seemed to interfere with the initial performance of animals, with the linear trend indicating a better performance of P301L-Tau mice at the baseline.

**Table 1:** **Descriptive statistics, correlations (below the diagonal), and variance/covariance (diagonal and above) matrix for the variables in the model**.

**Variable**	**1**	**2**	**3**	**4**	**5**	**6**	**7**	**8**	**9**	**10**	**11**	**12**	**13**
1. Day_1	145.200	29.953	31.046	15.028	30.426	17.677	34.941	27.835	16.681	–0.241	–0.448	–0.301	0.135
2. Day_2	0.174^*^	202.984	84.421	62.232	70.310	49.706	35.225	40.035	36.080	–0.469	0.206	0.528	–0.958
3. Day_3	0.180^*^	0.414^**^	204.533	90.804	74.053	45.415	64.316	55.841	68.906	0.221	0.031	0.521	–1.175
4. Day_4	0.083	0.292^**^	0.425^**^	223.110	112.522	75.203	61.031	51.030	62.034	−0.809	0.578	0.506	–0.862
5. Day_5	0.167^*^	0.327^**^	0.343^**^	0.499^**^	228.089	100.215	106.451	81.857	89.806	–1.014	0.752	0.374	–0.425
6. Day_6	0.101	0.240^**^	0.219^**^	0.346^**^	0.457^**^	211.193	112.201	87.525	73.582	–0.021	0.896	0.151	–0.001
7. Day_7	0.202^**^	0.172^*^	0.314^**^	0.285^**^	0.492^**^	0.538^**^	205.567	104.440	101.858	0.194	1.312	0.512	0.085
8. Day_8	0.171^*^	0.208^**^	0.289^**^	0.253^**^	0.401^**^	0.446^**^	0.539^**^	182.470	121.541	1.075	0.718	0.035	0.191
9. Day_9	0.103	0.188^*^	0.357^**^	0.308^**^	0.441^**^	0.375^**^	0.527^**^	0.667^**^	181.870	1.373	1.627	–0.121	0.086
10. Sex^a^	–0.040	–0.066	0.031	–0.108	–0.134	–0.003	0.027	0.159^*^	0.203^**^	0.251	0.000	0.004	0.018
11. Age^b^	–0.076	0.030	0.004	0.079	0.102	0.126	0.187^*^	0.109	0.247^**^	–0.002	0.239	–0.020	0.027
12. Stress^c^	–0.050	0.074	0.073	0.068	0.049	0.021	0.071	0.005	–0.018	0.015	–0.082	0.250	–0.009
13. Genotype^d^	0.022	–0.134	–0.164^*^	–0.115	–0.056	0.000	0.012	0.028	0.013	0.072	0.109	–0.036	0.251
Mean (or %)	43.25	40.86	37.02	35.66	32.85	31.28	29.46	25.83	24.10	49.2%	47.0%	61.2%	51.9%
SD	12.05	14.25	14.30	14.94	15.10	14.53	14.34	13.51	13.49	9696	9696	–	–

* *p < 0.05*;

** *p < 0.001*.

a *0 = Male, 1 = Female*.

b *0 = Mid-aged, Aged*.

c *0 = Control, 1 = Stressed*.

d *0 = Wild Type, 1 = Transgenic*.

### Comparison of statistical procedures

As this study, focuses on a detailed evaluation of learning progression (growth) and how it could be affected by different factors (namely aging, sex, environmental stress and genotype), a comparison of results from a classical Mixed-Design Factorial ANOVA and a combined Auto-Regressive/Latent Growth approach was performed.

The MDF ANOVA revealed a significant between-subjects effect of stress [*F*_(1, 167)_ = 4.87, *p* = 0.029, partial η^2^ = 0.028] and a sex * genotype interaction on the learning curve [*F*_(1, 167)_ = 7.34, *p* = 0.007, partial η^2^ = 0.042]. With respect to within-subjects effects, significant results were obtained for sex * day [*F*_(7.8, 1299.8)_ = 2.57, *p* = 0.010, partial η^2^ = 0.015] and stress * day [*F*_(7.8, 1299.8)_ = 2.25, *p* = 0.029, partial η^2^ = 0.013]. Regarding the ALT analysis, it was observed that sex significantly impacted both the baseline performance (intercept; CR = –2.19, *p* = 0.029, females presenting higher mean latencies) as well as the growth (slope; CR = 3.41, *p* < 0.001, females having decreased learning growth throughout time); stress produced a significant effect on learning growth (CR = 3.29, *p* = 0.001, stressed animals with reduced growth); and a stress * genotype interaction significantly affected the intercept (CR = 2.02, *p* = 0.043). Moreover, the ALT approach reveals a small positive correlation between Intercept (baseline levels) and Slope (learning growth), indicating that animals with higher initial scores undergo major changes, and animals with lower initial scores present smaller changes, although the significance scores were not statically relevant^3^. The summary of the main differences between statistical analyses is presented on Table 2.

**Table 2:** **Comparison of significant effects for individual factors and interactions obtained with Mixed-Design Factorial ANOVA and ALT procedures**.

	**MDF ANOVA**		**ALT**	
	**BS**	WS * Day	**ICEPT**	**SLOPE**
**FACTOR**				
Sex	ns	*p* < 0.05	*p* < 0.05	*p* < 0.05
Age	ns	ns	ns	ns
Stress	*p* < 0.05	*p* < 0.05	ns	*p* < 0.05
Genotype	ns	ns	ns	ns
**INTERACTIONS 2 FACTORS**				
Sex * age	ns	ns	ns	ns
Sex * stress	ns	ns	ns	ns
Sex * genotype	*p* < 0.05	ns	ns	ns
Age * stress	ns	ns	ns	ns
Age * genotype	ns	ns	ns	ns
Stress * genotype	ns	ns	*p* < 0.05	ns
**INTERACTIONS 3 FACTORS**				
Sex * age * stress	ns	ns	ns	ns
Sex * age * genotype	ns	ns	ns	ns
Sex * stress * genotype	ns	ns	ns	ns
Age * stress * genotype	ns	ns	ns	ns
**INTERACTION 4 FACTORS**				
Sex * age * stress * genotype	ns	ns	ns	ns

*BS, between-subjects; WS, within-subjects; ICEPT, Intercept; MDF ANOVA, Mixed-Design Factorial ANOVA; ALT, Auto-regressive Latent Trajectory*.

### Integrated ALT approach

**Figure 3:**
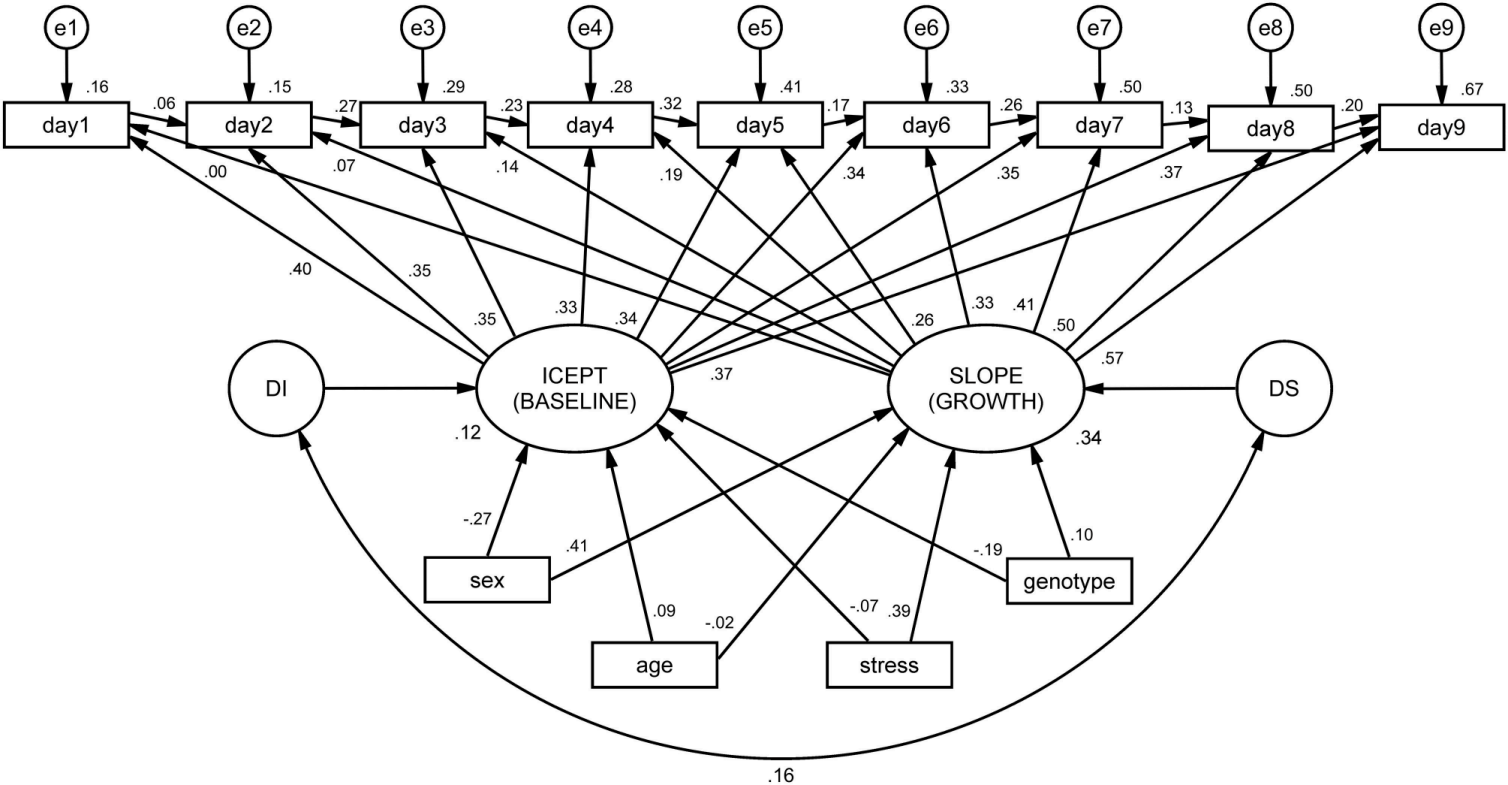
**ALT model for mean latencies on the Morris water maze (MWM), conditioned by sex, age, stress and genotype**. Squares and circles represent observed and unobserved (latent) variables, respectively. Observed variables “day1” to “day9” represent the individual latencies for each day of the MWM test. The arrows linking these variables form the auto-regressive subpart of the ALT approach (these can be interpreted as regression coefficients). Variables “e1” to “e9” represent measurement error terms for each acquisition day. These measurement errors correct the measured variances for random error. “ICEPT” (intercept) represents animals' baseline performance (estimated by the linear growth). In a Cartesian coordinate system, this variable represents the value of y when x is zero. In ALT models, it provides information about the sample mean and variance of the collection of intercepts that characterize each animal's latencies. The “SLOPE” represents the linear evolution of the latencies for each animal throughout time. “DI” and “DS” (D stands for disturbances) represent the variance of the intercept and slope, respectively. At the bottom, intrinsic and extrinsic factors are represented to observe their influence on the intercept and slope.

The integrated ALT model (Figure 3) revealed excellent fit indices [$\chi_{\left(45\right)}^{2}=60$.1, *p* = 0.435, CFI = 0.998, TLI = 0.997, RMSEA (HI90) = 0.010 (*p* = 0.968)]. The variances of both the intercept (latencies at the baseline) and the slope (growth during time) were significant (*p* < 0.001), indicating that the variance estimates for these parameters are significantly different from zero. The correlation between baseline latencies (intercept) and growth (slope) is not significant, suggesting that animals that display higher latencies at baseline do not differ in terms of growth from those with lower latencies. In other words, the cognitive performance of animals at day 1 (day 1 latency) is not a determinant factor for learning growth (slope). It was observed that both age and stress condition significantly affected the initial level, with old animals (B = 2894.86, SE = 4.73, *p* < 0.001) and stressed animals (B = 2903.17, SE = 417.10, *p* < 0.001) being both associated with increased mean latencies at first day of performance (baseline).

## Discussion

The simplicity of water mazes constructs is associated with their widespread use in assessment of memory and learning (Vorhees and Williams, 2014). Paradoxically, the interpretation of animal behavior in these mazes is complex. For instance, locomotion deficits and the adoption strategies unrelated with the paradigm (e.g., random swimming Whishaw and Mittleman, 1986) can lead to erroneous conclusions. In addition, intrinsic (e.g., strain, sex, age) and extrinsic (e.g., stress, drugs) factors have to be computed together with behavioral parameters (animals' performance at beginning, learning ability/growth and ceiling/floor limits) in robust statistical models. Aiming to provide an adequate tool for analysis of complex design experiments involving longitudinal testing and to compare it to traditional analysis, we implemented a comparative analysis to study the effects of different factors on animals' learning curve during nine acquisition days on the MWM paradigm, in which a SEM ALT model was contrasted with a MDF ANOVA. We found that in both procedures sex and stress had significant impact on the learning curve. Nevertheless, with the MFD ANOVA, it was only possible to compare groups on the average scores during acquisition days. The ALT approach extends the amount of information that can be extracted, allowing to disentangle group effects on different phases (baseline performance vs. learning growth). Specifically, MDF ANOVA indicated that both sex and stress produced significant within-subjects' effects. On the other hand, the ALT approach revealed that whereas sex produced a significant influence both to the basal levels and to the learning growth, stress produced a significant influence only on the learning growth. Thus, ALT revealed to more accurately differentiate the impact of individual characteristics to the learning process.

With respect to interaction effects, it was observed that sex * genotype had significant impact on between-subjects' effects in the traditional analysis that was not observed with the ALT approach. On the other hand, a significant stress ^*^ genotype effect was found on baseline performance, using the ALT approach. Thus, the ALT method has the advantage of considering animals' individual trajectories, compared to MDF ANOVA which only takes into account group means. For instance, considering two time-points, a subject with a score of 10 at baseline and 20 at the follow-up will obtain an average score of 15; as it will a subject with a score of 20 at baseline and 10 at the follow-up, even though their evolution occurs in opposite directions (see Table 3 for a comprehensive comparison on the models).

**Table 3:** **Comparison between Mixed-Design and ALT features**.

**Characteristics**	**MFD ANOVA**	**ALT**
Complexity	Moderate	High
Assumptions	Stricter assumptions (e.g., normal distribution, homogeneity of variances)	Less strict assumptions
Sample size required	Reduced^*^	>50
Type I error probability	High	Low
Interpretation	Difficult	Straightforward
Factors' weighing	Limited	Extensive
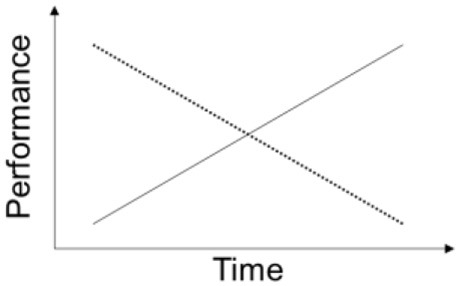	Does not capture between groups effects	Disentangles interaction effects
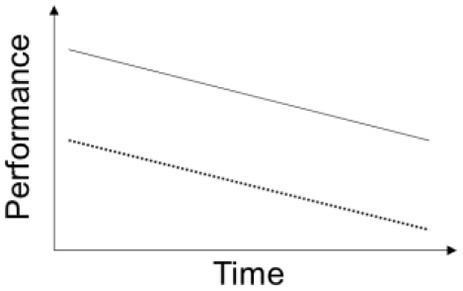	Assumes between group differences even if the growth is similar between groups	Captures similar growth

* *Although, general guidelines recommend a minimum sample size (n > 50) for the use of SEM-based approaches, the statistical power obtained is generally higher than traditional analyses above this threshold*.

To assess the total explained variance of both baseline performance and learning growth and to account for the shared variance between factors (which is not observed in typical Analyses of Variance), we have specified a model in which all factors were entered simultaneously. With this approach, we were able to explain 34% of animals' learning curve, with sex exerting a significant effect on both baseline performance and learning growth (females started with better performance, but learnt less than males), and stress significantly affecting the learning curve (stressed animals presented a decreased learning growth). The total explained variance is satisfactory, considering the heterogeneity between animals.

Based on our results, animal research may benefit from the use of this ALT approach, which allows a more comprehensive study of learning curves and other temporal patterns in tests like the MWM, compared to classical procedures, such as Mixed-Design Factorial ANOVA. This approach allows extensions of the MDF-ANOVA method (Duncan et al., 2006). It provides flexibility to assess measurement change, such as the accommodation of measurement error, the representation of different growth patterns and the establishment of cause-effect relationships on variables. With this approach, researchers are able to gain additional information, such as the influence of baseline performance on the performance during sessions. Also, besides addressing the influence of external factors, such as age or sex, on the animals' performance, it is possible to distinguish whether this influence is significant at the baseline or during the growth throughout trials. Altogether, this allows to extend the amount of information that can be extracted from statistical procedures, useful in biological significance.

Whereas MDF ANOVA allows modeling both the change over time and group differences in growth, it provides limited information about growth trajectories. Specifically, traditional procedures assume that change is linear and constant across time. In contrast, ALT allows to study both linear and non-linear growth patterns. Besides this, when using traditional procedures, it is presumed that measurement occurs without error, whereas ALT considers the measurement of error in the definition of the model. Also, classical ANOVA procedures require strong assumptions that are not frequently met in behavioral research, such as sphericity and/or homogeneity of variance/covariance, which can be easily accommodated with the approach herein presented (Hair et al., 2006). In addition, results from simulation studies revealed that SEM procedures developed to study learning growth require considerable less sample size to achieve comparable statistical power, when comparing to ANOVA traditional approaches (Fan, 2003). In fact, using a classical approach, it was demonstrated that there were significant interactions between stress and learning over time, with stressed animals presenting considerably worse performance in the MWM task (Sotiropoulos et al., 2015). With the ALT method, we were able to observe that the stress effects were particularly relevant for the learning growth, but not on baseline performance. Therefore, the use of ALT allows researchers to increase the complexity in the representation of learning growth and correlates of change. This strategy enables researchers to address both causal and consequential effects that may influence growth trajectory patterns (Fan, 2003).

ALT constitutes therefore a comprehensive approach to analyze growth and behavioral processes and it may be implemented not only in MWM and other water maze paradigms (working memory and egocentric referenced memory), but also in other behavioral paradigms such as the variable delay-to-signal (impulsivity Leite-Almeida et al., 2013), the 5-choice serial reaction time task (sustained attention Bari et al., 2008) and the risk-based decision-making (Morgado et al., 2014).

There are, nevertheless, some drawbacks associated with the proposed approach. For instance, one may discuss the adequacy of the sample size for conducting the ALT approach, since model fit parameters are dependent on the sample size, being more fluctuant on small samples. By performing Monte Carlo simulations, Hamilton and colleagues showed that sample sizes of at least 100 are recommended to reduce the likelihood of producing biased parameters. Nonetheless the authors recognize that samples above 50 yield model convergence (Hamilton et al., 2003). Another aspect is associated with the complexity of this analytical procedure, which requires continuous adjustments to the model to enhance fit indexes when compared to the traditional approach. Main differences between the two approaches are highlighted on Table 3.

In sum, taking into consideration the comparison between procedures herein conducted, we argue that statistical analysis of animal longitudinal experiments may benefit from the use of SEM-based approaches. These comprise a more comprehensive approach to the complex and temporal evolution of cognitive processing and overall behavioral performance.

## Author contributions

IS, JS, and AT conducted/supervised the behavioral experiments; PM, IS, JS, HL, and PC prepared and analyzed the behavioral database; PM and PC performed the statistical analyses; PM, IS, HL, NS, and PC prepared the manuscript. All authors contributed to the final/submitted version of the work.

### Conflict of interest statement

The authors declare that the research was conducted in the absence of any commercial or financial relationships that could be construed as a potential conflict of interest.

## Footnotes

^1^Latent variables represent unobserved constructs reflecting one or more observed variables. These variables are typically used in Structural Equation Modeling analyses as a strategy to assess the relationship between latent constructs. In the particular case of the LGM sub-model, two latent variables (intercept and slope) are defined to represent the baseline levels (estimated by the growth linear regression) as well as the evolution throughout time of the animals' latencies.

^2^Model fit is a comparison between the theory and the observed reality, through the assessment of the similarity of the estimated covariance matrix (i.e., the theory) to the observed covariance matrix (i.e., the reality). Specifically, the chi-square statistic (χ^2^) constitutes the fundamental measure for a mathematical comparison of the two matrices. Using the statistical significance of the χ^2^, we test the null hypothesis that the observed sample and the estimated covariance matrices are equal. That is, unlike other parametrical tests (in which we look for small *p*-values to demonstrate the existence of a significant relationship), in SEM-based analysis, a significant χ^2^ demonstrate that the two covariance matrices are statistically different, indicating a poor model fit. Nevertheless, the significance of this measure is influenced by both the sample and the number of variables included in the model. For this reason, researchers typically rely on additional fit measures that are less sensitive to the sample size. One of the most widely used measures is the Root Mean Square Error of Approximation (RMSEA), which attempts to correct for the penalization of large samples and/or complex models. Both of these measures are absolute fit indices, meaning that the model is tested independently of other possible models. Other measures compare how the estimated model fits in comparison to baseline models (models in which all observed variables are uncorrelated)—incremental fit indices. These measures indicate how well the establishment of relationships between variables contribute to a better representation of data. Considering that different fit measures reflect different properties, it is generally advised the use of the χ^2^ statistic together with an incremental (such as the CFI) and another absolute index (such as the RMSEA) (Hair et al., 2006).

^3^In order to demonstrate that our findings were not restricted to the analysis of latencies, we tested this model for the analysis of distances to reach the central platform with comparable results being obtained. We observed that, despite the fact that the most relevant factors were identified with both procedures, the ALT approach enabled the differentiation of the stage of the learning curve that were significantly affected.
